# Trends in admission rates of primary angle closure diseases for the urban population in China, 2011–2021

**DOI:** 10.3389/fpubh.2024.1398674

**Published:** 2024-06-05

**Authors:** Qi Chen, Peng Shen, Mengtian Zhou, Yang Cao, Xuanli Zheng, Fengping Zhao, Haishuang Lin, Yutong Ding, Yiting Ji, Jingjing Zuo, Hongbo Lin, Yuanbo Liang

**Affiliations:** ^1^National Clinical Research Center for Ocular Diseases, Eye Hospital, Wenzhou Medical University, Wenzhou, China; ^2^Department of Ophthalmology, The Second Affiliated Hospital of Zhengzhou University, Zhengzhou, China; ^3^Yinzhou District Center for Disease Control and Prevention, Ningbo, China; ^4^Glaucoma Research Institute, Wenzhou Medical University, Wenzhou, China

**Keywords:** primary angle closure diseases, cataract surgery, laser peripheral iridotomy, regional health big data, trends

## Abstract

**Background:**

Cataract surgery and laser peripheral iridotomy (LPI) are effective approaches for preventing primary angle closure diseases (PACDs), as well as acute primary angle closure (APAC). Due to the development of population screening and increases in cataract surgery rates, this study aimed to examine trends in the admission rates of PACD among the urban population in China.

**Methods:**

This cross-sectional study examined patients who were admitted to a hospital for PACD, and who underwent cataract surgery or LPI operations. The data were obtained from the Yinzhou Regional Health Information Platform (YRHIP) from 2011 to 2021. The annual rates of PACD and APAC admissions, cataract surgery and LPI were analyzed, with the number of cases used as numerators and the annual resident population in Yinzhou district used as denominators.

**Results:**

A total of 2,979 patients with PACD admissions, 1,023 patients with APAC admissions, 53,635 patients who underwent cataract surgery and 16,450 patients who underwent LPI were included. The number of annual admissions for PACD gradually increased from 22 cases (1.6/100000) in 2011 to 387 cases (30.8/100000) in 2016, after which it decreased to 232 cases (16.2/100000) in 2019 and then increased to 505 cases (30.6/100000) in 2021. The number of cataract surgeries gradually increased from 1728 (127.7/100000) in 2011 to 7002 (424.9/100000) in 2021. Similarly, the number of LPI gradually increased from 109 (8.0/100000) in 2011 to 3704 (224.8/100000) in 2021.

**Conclusion:**

The admission rates of PACD for the urban population in China have declined in recent years after a long increasing trend in the rates of cataract surgery and LPI. However, it increased rapidly during the COVID-19 epidemic. The national health database should be further utilized to investigate temporal trends in the prevalence of PACD.

## Introduction

Glaucoma is the most common irreversible and age-related blinding eye disease in the world. In 2020, there were 76.0 million people with glaucoma, and this number is expected to increase to 111.8 million by 2040 (1). It has also been reported that primary glaucoma differs across regions, ages, sexes and ethnicities (2). Based on the angle status and pathogenesis, glaucoma is classified as open angle glaucoma (OAG) or angle-closure glaucoma (ACG). The ACG possesses a 3-fold greater risk of severe, bilateral visual impairment than does the OAG (3). Moreover, the number of PACGs is the largest in Asia (1, 4). This difference is mainly due to the high prevalence in China (5–10), which has an estimated 28 million primary angle closure suspects (PACSs), 9 million people with primary angle closures (PACs) and 4.5 million people with PACG (11).

In recent decades, a series of epidemiological studies on glaucoma have been conducted (5–10). These surveys have revealed the characteristics and distribution of glaucoma in China, thereby providing a meaningful reference for glaucoma prevention and treatment. However, traditional population-based studies have mainly adopted sample surveys; in such studies, the confidence intervals for prevalence are strongly affected by both the sample size and the response rate. In addition, the small population coverage, various population compositions and inconsistent diagnostic criteria make it impossible to directly assess trends in glaucoma prevalence over time.

With the application of big data analysis in medical areas, large population-based health administrative databases are rapidly developing into abundant resources for ophthalmic research, such as disease surveillance and health service utilization (12, 13). A previous Taiwanese study (14) demonstrated a negative relationship between the rates of cataract surgery and acute primary angle closure (APAC) admissions based on the National Health Insurance Research Database between 1997 and 2004. Studies in England (15) using the 1968–2004 health administrative database and studies in Scotland using the 1998–2012 national database suggested that the rates of cataract surgery may decrease the number of PACGs (16). However, these studies were performed early (14–17), and the results fail to represent the current situation of health care utilization and disease prevalence in China.

Angle closure is fundamental in the pathogenesis of PACG. Although PACG is irreversible, it can be prevented or controlled early. The goals of managing primary angle closure diseases (PACDs) are to reverse or prevent angle closure and control intraocular pressure, thereby preventing optic neuropathy. Cataract surgery and laser peripheral iridotomy (LPI), which are the treatments recommended by glaucoma guidelines (18–20), have been widely shown to open angles and effectively relieve pupillary block. Furthermore, cataract surgery rates (CSRs) have increased significantly in recent decades in China. Therefore, we examined the trends in admission rates of PACD patients by using Yinzhou Regional Health Information Platform (YRHIP) from 2011 to 2021 and compared these rates with rates of cataract surgery or LPI. The results of this study can help health policymakers in implementing adequate and sustainable prevention strategies.

## Methods

The Ethics Committee of the Eye Hospital of Wenzhou Medical University (Wenzhou, China) approved this study before its commencement, and this study was performed in accordance with the principles outlined in the Declaration of Helsinki. Informed consent was waived because the data were deidentified and retrospectively collected. Similarly, the informed consent waiver was approved by the Ethics Committee of the Eye Hospital of Wenzhou Medical University.

### Patients

Patients who were discharged with PACD and who received antiglaucoma treatment (medicine, laser, or surgery), patients who underwent cataract surgery according to surgical records, and patients who underwent LPI were included in our study. Patients who were ≤ 40-years-old at diagnosis or at treatment were excluded from the study.

### Yinzhou regional health information platform

Yinzhou District is a highly developed economy in Ningbo city, Zhejiang Province, China, with a population of more than 1.6 million reported in 2020 (21). The Yinzhou regional health information platform (YRHIP) was developed by the Yinzhou District Health Bureau in 2005 based on electronic health records and was completed in 2010. It covers all level 1 and above hospitals of the whole district, as well as their affiliated community health care centers and township health centers; moreover, it realizes the sharing of medical health information between the Centers for Disease Control and Prevention, comprehensive hospitals and community health care institutions. The YRHIP covers almost all health-related activities of residents from birth to death. Currently, 95% of the resident population is covered by this platform (22), which integrates public health, medical services, and electronic medical records (EMRs). The data are relatively complete and recorded in real time, thus ensuring the reliability of this study.

### Standard definition

The PACDs in this study included PACS, PAC, and PACG (23). PACS was defined as the presence of ≥ 180° appositional contact between the peripheral iris and posterior trabecular meshwork. PAC was defined as the presence of ≥ 180° occludable drainage angle and trabecular obstruction by the peripheral iris combined with elevated intraocular pressure (IOP) or peripheral anterior synechiae (PAS). PAC accompanied by glaucomatous optic neuropathy (GON) was classified as PACG. APAC is a subgroup of PACD and is characterized by an acute increase in IOP, with typical symptoms of acute vision loss, intense ocular pain or systemic symptoms, including nausea, vomiting, or headache ipsilateral to the affected eye(s). As one of the most common diseases in the ophthalmic emergency department, it is necessary to rapidly decrease the intraocular pressure of APAC patients to control further progression of the disease (24).

### Quality control and data collection

1. To ensure the accuracy of the diagnosis, we included inpatients in which the primary diagnosis at discharge was PACD.
2. If ACD was not specified as the primary or secondary diagnosis, we linked the diagnosis information of outpatients and inpatients through personal unique identification codes (desensitization). PACD was diagnosed when patients did not present with secondary causes such as neovascularization, uveitis, trauma or lens-related causes.
3. To avoid omissions, inpatients without a primary diagnosis of PACD but who underwent antiglaucoma treatment during hospitalization were included.
4. If the subtype of PACD was not specified (acute or chronic), patients who were diagnosed with APAC according to the hospitalization fee information, including at least one anterior chamber puncture, methazolamide or 20% mannitol, were included.

The data on PACD admissions, cataract surgery and LPI operations were retrospectively retrieved from the EMRs of the YRHIP between 2011 and 2021. Data extraction was performed using the database management software “Hive”. The data of a patient with multiple admissions for the same diagnosis or operation within 1 year were calculated once.

### Statistical analysis

Continuous variables with a normal distribution and skewed distribution are expressed as the mean ± standard deviation and median (P25, P75), respectively. Categorical variables are expressed as frequencies (%). The Bureau of Statistics in Yinzhou District disclosed the total resident population every year. The annual admission rates of PACD, APAC and non-APAC patients and the annual rates for cataract and LPI surgeries were calculated, with the number of cases used as numerators and the annual resident population in Yinzhou district used as denominators. These rates were stratified by age group (40–49, 50–59, 60–69, 70–79 and ≥80 years) and sex. Statistical analysis was performed with Statistical Product Service Solutions (SPSS, IBM Corporation) software 24.0, and a *p* value < 0.05 was considered to indicate statistical significance.

## Results

**Figure 1:**
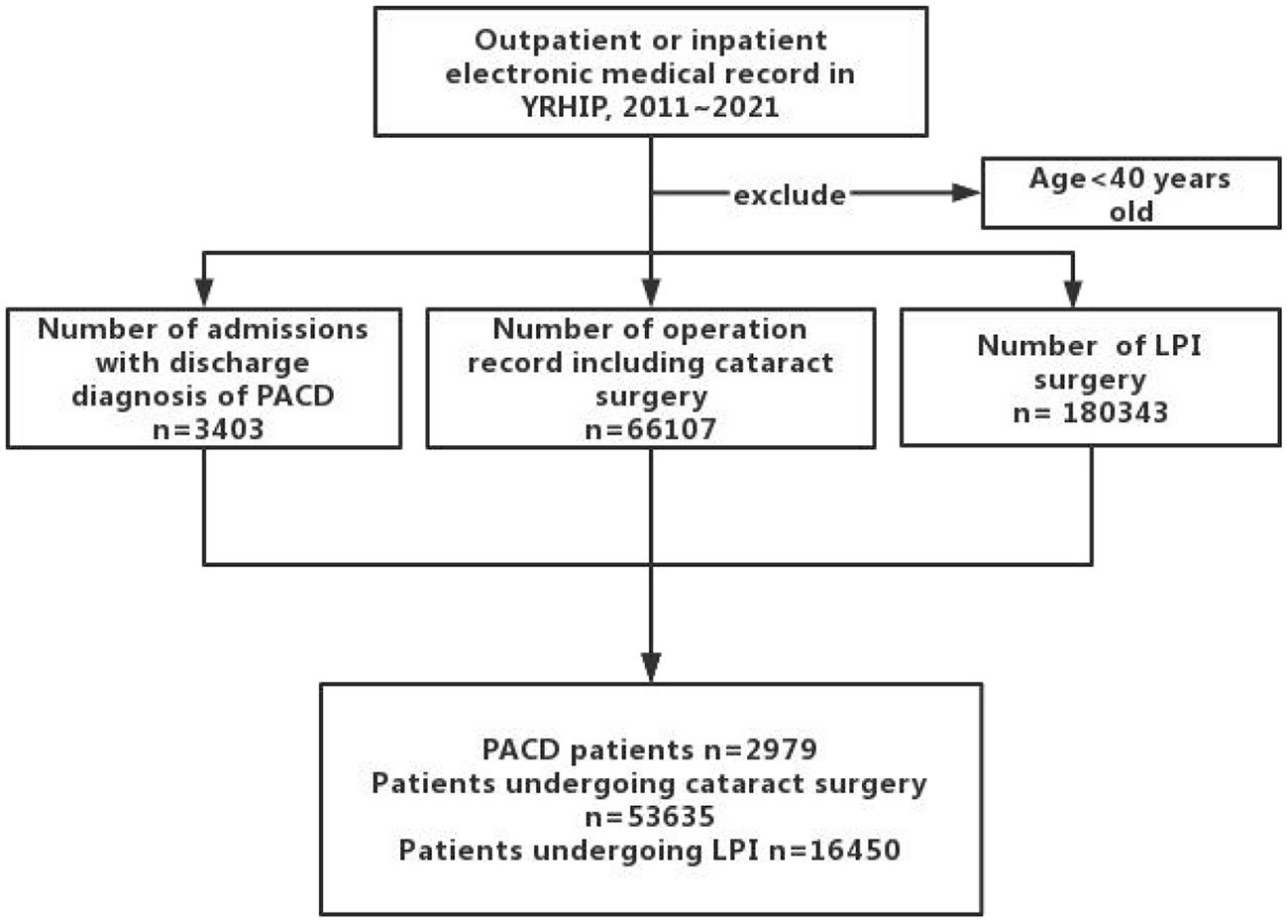
Inclusion and exclusion diagram of the study participants.

A total of 2,979 patients (897 males and 2,082 females) with PACD, 1,023 patients (238 males and 785 females) with APAC, 53,635 patients (22,094 males and 31,541 females) who underwent cataract surgery and 16,450 patients (6,732 males and 9,718 females) who underwent LPI in the Yinzhou District between 2011 and 2021 (Table 1 and Figure 1) were included. The median age of patients with PACD was 67 years (interquartile range [IQR] 61–74 years), and the median ages of patients with APAC and non-APAC were 68 years (IQR 61–74 years) and 67 years (IQR 61–73 years), respectively. The median age of patients who underwent cataract surgery was 70 years (IQR 63–76 years), and that of patients who underwent LPI was 60 years (IQR 51–68 years).

**Table 1:** Demographic characteristics of the subjects included in the study.

**Variable**	**APAC admissons**		**PACD admissions**		**Cataract surgery**		**LPI**	
	**Total no**	**%**	**Total no**.	**%**	**Total no**.	**%**	**Total no**.	**%**
**Age group (years)**								
40–49	30	2.9	114	3.8	2,247	4.2	3,260	19.8
50–59	184	18.0	517	17.4	6,392	11.9	4,671	28.4
60–69	375	36.7	1,158	38.9	17,880	33.3	5,136	31.2
70 79	317	31.0	895	30.0	18,789	35.0	2,578	15.7
80+	117	11.4	295	9.9	8,327	15.5	805	4.9
**Gender**								
Male	238	23.3	897	30.1	22,094	41.2	6,732	40.9
Female	785	76.7	2,082	69.9	31,541	58.8	9,718	59.1

### Annual rates of PACD admissions, cataract surgery and LPI

**Figure 2:**
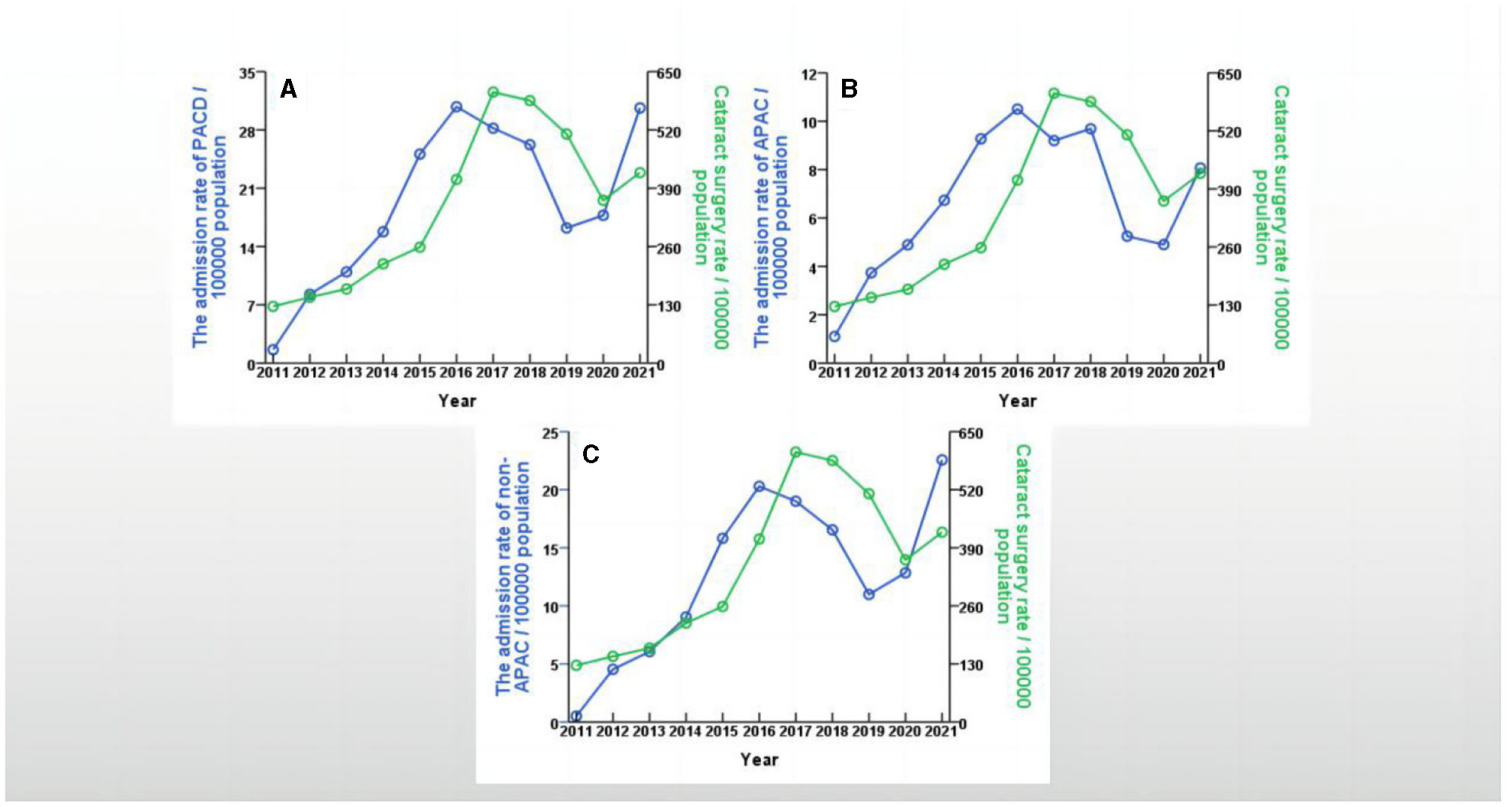
Trends in the annual rates between PACD admission, APAC admission, non-APAC admission and cataract surgery in Yinzhou District, Ningbo city, China, 2011–2021. **(A)** showed trends in the annual rates between PACD admission and cataract surgery in Yinzhou District, 2011–2021; (B) showed trends in the annual rates between APAC admission and cataract surgery in Yinzhou District, 2011–2021; **(C)** showed trends in the annual rates between non-APAC admission and cataract surgery in Yinzhou District, 2011–2021.

**Figure 3:**
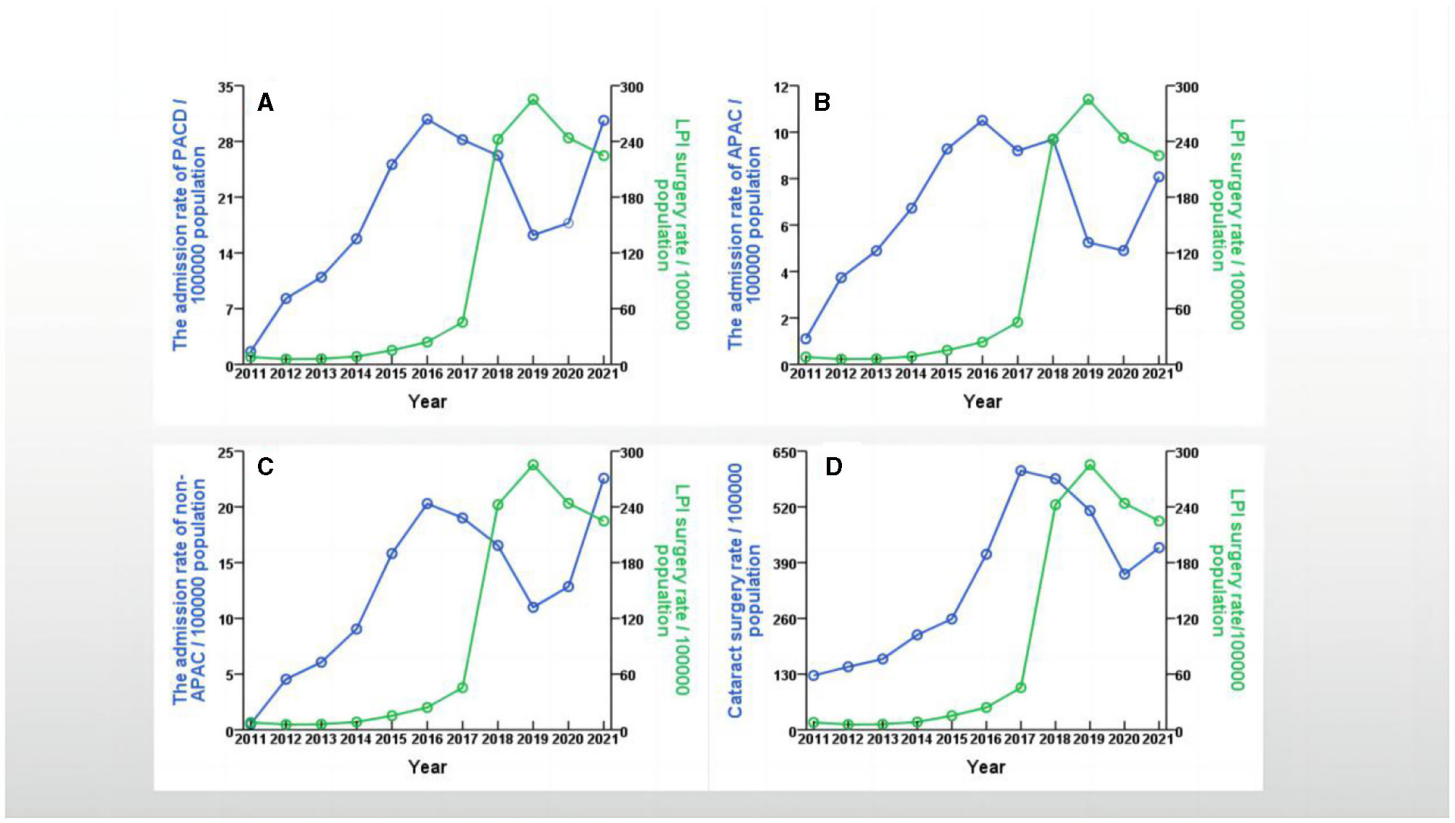
Trends in the annual rates between PACD admission, APAC admission, non-APAC admission, cataract surgery and LPI in Yinzhou District, Ningbo city, China, 2011–2021. **(A)** showed trends in the annual rates between PACD admission and LPI in Yinzhou District, 2011–2021; **(B)** showed trends in the annual rates between APAC admission and LPI in Yinzhou District, 2011–2021; **(C)** showed trends in the annual rates between non-APAC admission and LPI in Yinzhou District, 2011–2021; **(D)** showed trends in the annual rates between cataract surgery and LPI in Yinzhou District, 2011–2021.

The number of annual admissions for PACD gradually increased from 22 cases (1.6/100000) in 2011 to 387 cases (30.8/100000) in 2016, decreased to 232 cases (16.2/100000) in 2019, and then increased rapidly to 505 cases (30.6/100000) in 2021. Similarly, the annual admissions for APAC gradually increased from 15 cases (1.1/100000) in 2011 to 132 cases (10.5/100000) in 2016, decreased to 75 cases (5.2/100000) in 2019, and then increased rapidly to 133 cases (8.1/100000) in 2021. Moreover, the number of cataract surgeries gradually increased from 1,728 (127.7/100000) in 2011 to 7,002 (424.9/100000) in 2021; the highest number of surgeries occurred in 2018 (7,856; 585.4/100000). The number of LPI operations gradually increased from 109 (8.0/100000) in 2011 to 4,077 (285.3/100000) in 2019 and then decreased to 3,704 (224.8/100000) in 2021 (Table 2). The trends in the annual rates of PACD admissions, APAC admissions, non-APAC admissions and cataract surgeries in the Yinzhou District are shown in Figure 2. The trends in the annual rates of PACD admission, APAC admission, non-APAC admission and LPI are shown in Figure 3.

**Table 2:** The annual rates of APAC and PACD admissions, cataract surgery and LPI operations in the Yinzhou district.

**Year**	**APAC admissons**		**PACD admissions**		**Cataract surgery**		**LPI**	
	**No**.	**Rates (/100000)**	**No**.	**Rates (/100000)**	**No**.	**Rates (/100000)**	**No**.	**Rates (/100000)**
2011	15	1.1	22	1.6	1,728	127.7	109	8.0
2012	51	3.7	113	8.3	2,006	146.9	77	5.6
2013	67	4.9	150	11.0	2,264	165.3	83	6.1
2014	93	6.7	218	15.8	3,060	221.4	117	8.5
2015	129	9.3	349	25.1	3,597	258.6	211	15.2
2016	132	10.5	387	30.8	5,148	409.5	302	24.0
2017	119	9.2	365	28.2	7,820	604.3	588	45.4
2018	130	9.7	352	26.2	7,856	585.4	3252	242.3
2019	75	5.2	232	16.2	7,303	511.1	4077	285.3
2020	79	4.9	286	17.7	5,851	363.0	3930	243.8
2021	133	8.1	505	30.6	7,002	424.9	3704	224.8

### Age-specific and sex-specific rates of PACD admission, cataract surgery and LPI

The age-specific rates of patients with PACD admissions, APAC admissions, and non-APAC admissions and the annual rates of patients who underwent cataract surgery and LPI stratified by age are shown in Supplementary Figure 1. The highest admission rates of PACD , APAC and non-APAC patients were observed in the 60–69-year-old age group, and the lowest rates were observed in the 40–49-year-old age group. The rates of cataract surgery were highest in the 70- to 79-year-old age group and lowest in the 40- to 49-year-old age group. Moreover, the rates of LPI were highest in the 60–69-year-old age group and lowest in the > 80-year-old age group. The sex-specific rates of patients with PACD admissions, APAC admissions, and non-APAC admissions and the annual rates of patients who underwent cataract surgery and LPI are shown in Supplementary Figure 2. The admission rates of PACD, APAC and non-APAC for females were higher than those for males, and the rates of cataract surgery and LPI for females were also greater than those for males.

The admission rates of PACD patients with non-acute attacks were always greater than those with acute attacks, as shown in Supplementary Figure 3A. The admission frequency of patients with PACD who experienced acute attacks gradually decreased over time, whereas that of patients with PACD who experienced nonacute attacks increased annually (Supplementary Figure 3B).

## Discussion

This study is the first to report the trends in the rates of PACD admissions, APAC admissions, non-APAC admissions, cataract surgeries and LPI operations from 2011 to 2021 for the urban population in China.

The results demonstrated that the rates of PACD admissions, APAC admissions, and non-APAC admissions increased from 2011 to 2016, after which they decreased significantly from 2017 to 2019. We speculated that the increased admission rates may be attributed to improvements in patients' eye health awareness and progress in disease diagnosis. In contrast, the increased CSR contributed to the decrease in the PACD and APAC admission rates. First, with population aging, an increase in life expectancy and population growth in China, the prevalence of age-related eye diseases has increased over time, and the disease spectrum has gradually changed from infectious diseases (24) to non-communicable diseases, including cataracts and glaucoma. Therefore, changes in population composition could not explain the decreased admission rates of PACD and APAC patients. Second, cataracts, which is the leading cause of blindness worldwide (25), represents a high-priority public health issue for the prevention and treatment of blindness and eye health in China, thus leading to the implementation of a series of national prevention and treatment projects for cataract blindness. The CSR was used as a proxy indicator for accessing cataract services in a country and was closely associated with economic indicators. It increased from 963 in 2011 to 3,143 in 2019. Previous studies (14–16) using national administrative databases have demonstrated a negative correlation between APAC admission rates and CSR. He et al. (26) simulated a significant increase in CSR from 2000 to 12000 based on data from the Liwan Eye study, which observed a remarkable decrease in the prevalence of ACG from 11.4% to 10.1%. Third, the lens is an important anatomical factor involved in the angle closure of PACD patients, and it progressively enlarges with age. Patients with PACD usually have particular anatomical characteristics, such as a small cornea, shallow anterior chamber, lens thickening, and hypermetropia. Moreover, research has shown that PACD also coexists with cataracts. Lowe (27) reported that increased lens thickness caused a shallow anterior chamber and advanced lens position. Both anatomical changes resulted in lens-iris diaphragm forward bulging, more extensive contact between the lens, the trabecular meshwork and the iris, and the consequent exacerbation of the pupillary block and angle closure. However, various studies (28–31) have shown that anterior segment parameters and peripheral anterior synechiae can significantly improve after phacoemulsification or combined phacotrabeculectomy in PAC or PACG patients. In 2016, Azuara-Blanco et al. (32) compared the efficacy, safety and cost-effectiveness of clear-lens extraction and LPI in the PAC and PACG groups, and their findings suggested that clear lens extraction was a preferred treatment. Finally, there may be a threshold effect and time delay in the effect of cataract surgery on PACD admission rates. Specifically, once the CSR exceeded a specific value, the rate of PACD admissions decreased. Thus, the gradual increase in CSR, especially the significant growth from 2015 to 2017, may have contributed to the decline in the PACD admission rates.

From 2020 to 2021, the rates of PACD admissions increased again, which we speculated was partly due to the community-wide lockdown, home confinement and increased social distancing during the COVID-19 pandemic. Confinement impedes people from promptly seeking medical services, such as conventional cataract surgery and LPI. Moreover, severe acute respiratory syndrome coronavirus 2 (SARS-CoV-2) infection causes hyponatremia (33) and changes in choroid thickness (34, 35), which may be predisposing factors for APAC. Moreover, insufficient prone position ventilation (36), poor sleep (37) and systemic drugs (38) can also induce angle closure.

Although there was no significant correlation between the rates of PACD admissions and LPI operations overall, we observed a strong inverse relationship after 2016 (*r* = −0.943, *p* < 0.01). The gradual increase in LPI surgery rates, especially from 2017 to 2019, may have led to a decrease in PACD admission rates. LPI can communicate and balance anterior and posterior chamber pressures to open the anterior angle chamber, and prophylactic LPI can reduce the risk of angle closure or acute glaucoma attack by 47% (39, 40). Thus, LPI was recommended as the first-line treatment for early PAC and PACG, except for patients with severe cataracts. The Zhongshan angle closure prevention (ZAP) clinical trial (39), which aimed to explore the efficacy and safety of LPI in preventing the transition of PACS to PAC, suggested that the transitional probabilities from PACS to PAC were extremely low and that the patients mainly experienced mild disease. It proposed against the widespread implementation of LPIs from the public health perspective. Although ZAP was a single-center randomized controlled trial, its conclusions cannot be generalized to the entire country. Clinical practice on prophylactic LPIs should be based on glaucoma guidelines and consider individual characteristics.

In addition, the annual rates of APAC admissions and cataract surgery were greater for females than for males, which was consistent with previous studies based on national health databases (14, 15). The age groups with the highest rates of APAC admissions and cataract surgery were younger than those mentioned in the British study (15). Finally, the admission frequency of patients with APAC gradually decreased over time. This phenomenon might be attributed to the prevention effects of cataract surgery and LPIs for PACD patients with acute attacks. So the increased rates of cataract surgery and LPI operations over time decreased the admission frequency of patients with APAC.

### Limitations

There were several limitations in our study. First, the findings in this study merely represented the urban population in southern China, and the findings may not be generalizable to the whole country due to variations in the genetic background, geographical environment, medical conditions and economic status between northern and southern China. Second, although YRHIP incorporates electronic medical records and claims data and covers the medical practices of district hospitals and clinics, the rates of PACD and APAC admissions, cataract surgery and LPI operations might be underestimated because local patients may seek medical services outside Yinzhou District, despite this probability being very low. Third, the study did not include outpatients with PACD, as we were interested in long-term trends in PACD admission rates. Compared with PACD outpatients, inpatients consumed more healthcare resources with higher medical expenses. The condition of hospitalized patients with PACD is more severe. Meanwhile, cataract surgery and LPI are effective approaches for preventing primary angle closure diseases (PACDs). Due to the development of population screening and increases in cataract surgery rates, the current study aimed to examine trends in the admission rates of PACD among the urban population in China.

## Conclusions

In conclusion, our study revealed that the admission rates of PACD for the urban population in China have started to decline in recent years after a long period of increase in the rates of cataract surgery and LPI operations. However, it grew rapidly during the COVID-19 pandemic. Moreover, we also found that the rates of PACD admissions, APAC admissions, cataract surgery and LPIs for females were greater than those for males. The admission rates of PACD and APAC were highest in the 60- to 69-year-old age group. Moreover, the rates of cataract surgery and LPI surgery were highest in the 70- to 79-year old and 60- to 69-year-old age groups, respectively. Finally, the national health database should be further utilized to investigate trends in the prevalence of PACDs over time.

## Data availability statement

The raw data supporting the conclusions of this article will be made available by the authors, without undue reservation.

## Ethics statement

The studies involving humans were approved by the Ethics Committee of the Eye Hospital of Wenzhou Medical University (Wenzhou, China). The studies were conducted in accordance with the local legislation and institutional requirements. Written informed consent for participation was not required from the participants or the participants' legal guardians/next of kin in accordance with the national legislation and institutional requirements.

## Author contributions

QC: Conceptualization, Formal analysis, Methodology, Writing – original draft, Writing – review & editing. PS: Data curation, Resources, Software, Writing – review & editing. MZ: Writing – review & editing. YC: Writing – review & editing. XZ: Writing – review & editing. FZ: Writing – review & editing. HL: Writing – review & editing. YD: Writing – review & editing. YJ: Writing – review & editing. JZ: Writing – review & editing. HL: Resources, Supervision, Writing – review & editing. YL: Funding acquisition, Project administration, Writing – review & editing.
