# Supplementary material for: Trends in admission rates of primary angle closure diseases for the urban population in China, 2011–2021
Source: Front Public Health. 2024 Jun 5;12:1398674. doi: 10.3389/fpubh.2024.1398674 (PMC11188465; doi:10.3389/fpubh.2024.1398674)
Supplement: Supplementary file 1 [file Data_Sheet_1.docx]

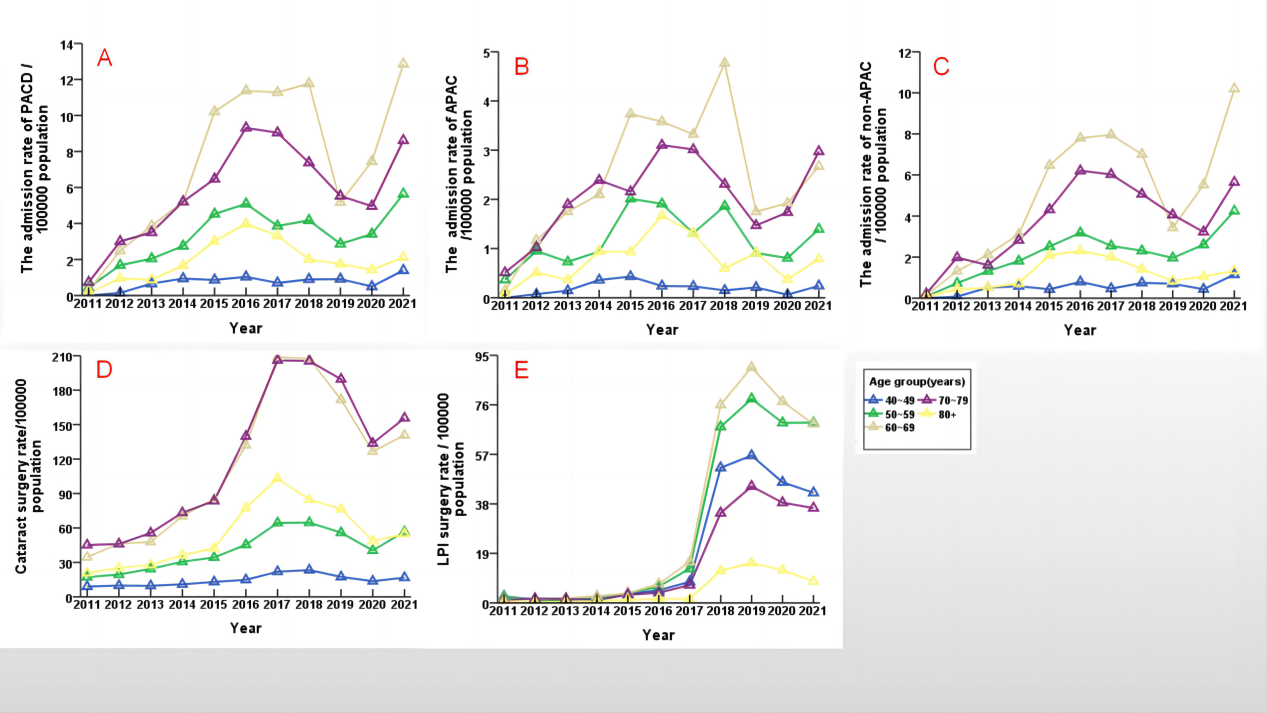


**Figure 1**. Annual rates of PACD admission, APAC admission, non-APAC admission, cataract surgery and LPI by age group in Yinzhou Distract, Ningbo city, China, 2011-2021


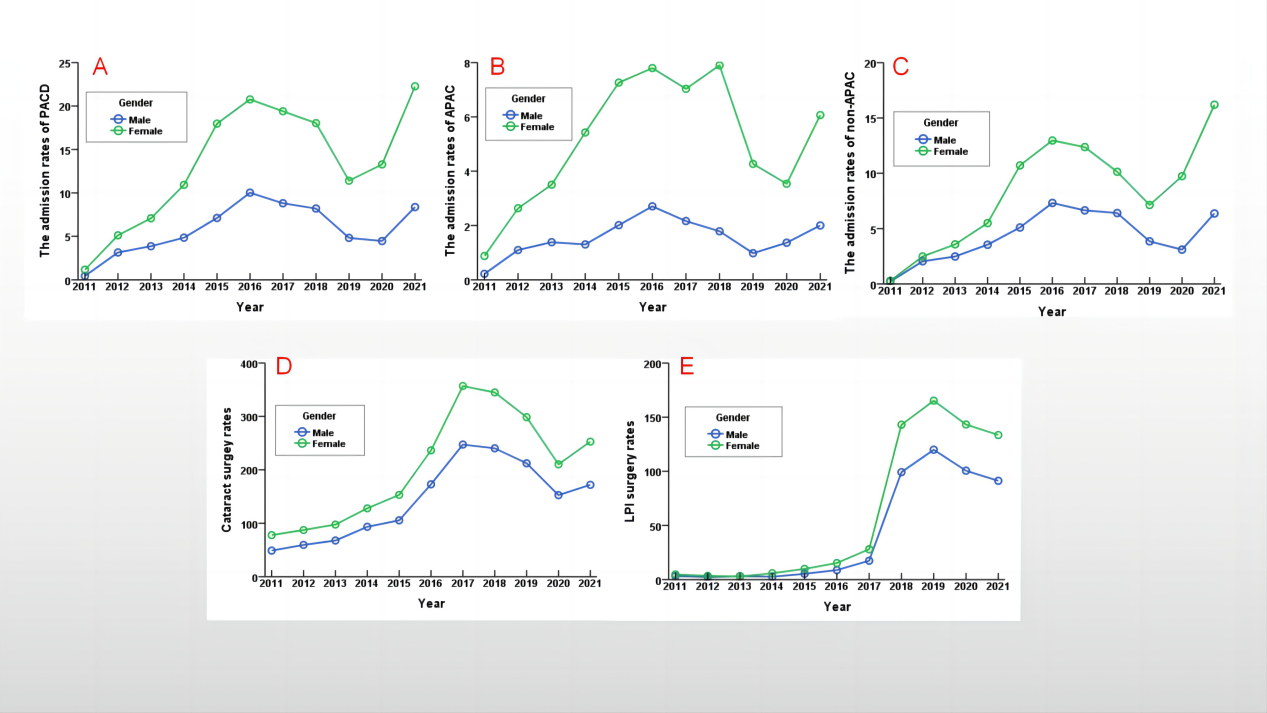


**Figure 2**. Annual rates of PACD admission, APAC admission, non-APAC admission, cataract surgery and LPI by gender in Yinzhou Distract, Ningbo city, China, 2011-2021


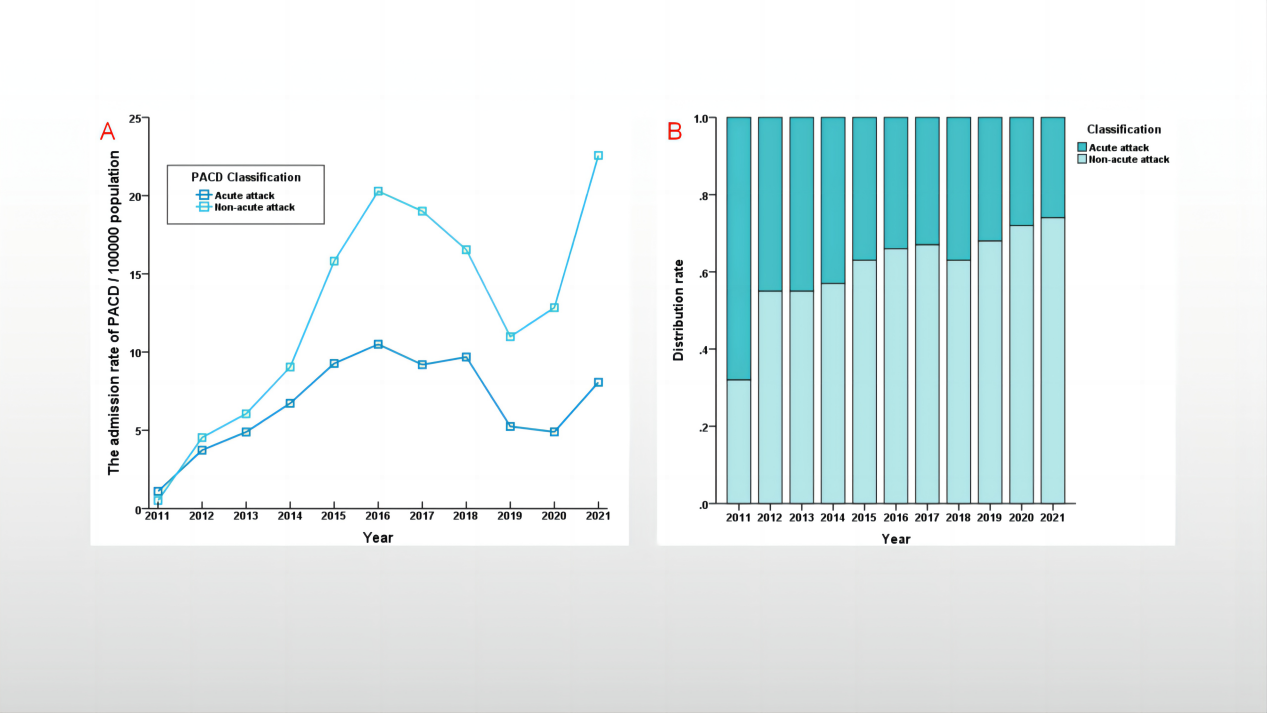


**Figure 3**. Annual admission rates and the distribution of admission frequency of PACD acute attack and non-acute attack in Yinzhou Distract, Ningbo city, China, 2011-2021
